# A novel double-antibody sandwich ELISA based on monoclonal antibodies against the viral spike protein detects porcine deltacoronavirus infection

**DOI:** 10.1128/spectrum.02854-24

**Published:** 2025-02-27

**Authors:** Yingjie Bai, Ruiming Yu, Guangqing Zhou, Liping Zhang, TianTian Wang, Ya Liu, Dongsheng Wang, Zhongwang Zhang, Yonglu Wang, Huichen Guo, Li Pan, Xinsheng Liu

**Affiliations:** 1 State Key Laboratory for Animal Disease Control and Prevention, College of Veterinary Medicine, Lanzhou University, Lanzhou Veterinary Research Institute, Chinese Academy of Agricultural Sciences, Lanzhou, China; 2 Gansu Province Research Center for Basic Disciplines of Pathogen Biology, Lanzhou, China; 3 College of Veterinary Medicine, Gansu Agricultural University 74661 , Lanzhou, China; Children's National Hospital, George Washington University, Washington, DC, USA

**Keywords:** porcine deltacoronavirus (PDCoV), spike protein, double-antibody sandwich enzyme-linked immunosorbent assay (DAS-ELISA)

## Abstract

Porcine deltacoronavirus (PDCoV) is a significant emerging pathogen that causes severe enteric disease in swine, and therefore significant economic losses in the pig farming industry. Here, we developed a novel double-antibody sandwich enzyme-linked immunosorbent assay (DAS-ELISA) based on two monoclonal antibodies directed against the PDCoV spike protein. These two monoclonal antibodies were obtained through hybridoma fusion and screening, and they can specifically react with the PDCoV spike protein. The detection limits of the DAS-ELISA for the recombinant spike protein and viral titer were approximately 0.12 ng/mL and 1.96 × 10³ copies/μL, respectively. The DAS-ELISA did not cross-react with other swine enteric coronaviruses, including porcine epidemic diarrhea virus, transmissible gastroenteritis virus, or porcine rotavirus. A total of 145 rectal swab samples were collected and tested for the presence of PDCoV with the DAS-ELISA and reverse transcription-quantitative PCR (RT-qPCR). The coincidence rate between the DAS-ELISA and RT-qPCR was 91.03%, with a kappa value of 0.814, indicating that the DAS-ELISA is a reliable method for viral antigen detection in clinical samples. DAS-ELISA had a sensitivity of 92.85% and a specificity of 89.89%. The positive predictive value and negative predictive value of this method are 85.25% and 95.24%, respectively. Furthermore, the DAS-ELISA can also be used to detect the spike protein in PDCoV vaccines, making it a valuable tool for assessing the efficacy of PDCoV vaccines.

**IMPORTANCE:**

Since 2014, porcine deltacoronavirus (PDCoV) has spread widely across multiple countries and regions, causing significant economic losses to the global livestock industry. Currently, no commercially available vaccine exists for the prevention of PDCoV infection; therefore, accurate and effective diagnostic methods are crucial for its control and prevention. In this study, the PDCoV S protein expressed in Chinese Hamster Ovary (CHO) cells was used to immunize mice, and a novel double-antibody sandwich enzyme-linked immunosorbent assay (DAS-ELISA) was established based on two monoclonal antibodies. The DAS-ELISA had high sensitivity, good repeatability, strong specificity, and high consistency for detecting clinical samples and spike protein in PDCoV vaccines. Therefore, the DAS-ELISA established in this study may be a reliable and effective tool for detecting PDCoV infection and the efficacy of PDCoV vaccines.

## INTRODUCTION

Porcine deltacoronavirus (PDCoV) is an enveloped, single-stranded positive-sense RNA virus with a genome length of approximately 25.4 kb, belonging to the order *Nidovirales*, family *Coronaviridae*, subfamily *Orthocoronavirinae*, and genus *Deltacoronavirus* (1, 2). Like porcine epidemic diarrhea virus (PEDV), transmissible gastroenteritis virus (TGEV), and porcine rotavirus (PoRV), PDCoV is an important intestinal pathogen of pigs, causing watery diarrhea, vomiting, dehydration, and high mortality in suckling piglets (3). However, compared with the other three viruses, the symptoms of PDCoV in finishing pigs and sows are relatively mild, the mortality rate is lower, and the affected pigs gradually regain their health. Generally, diarrhea symptoms can be observed within 20–48 h of viral infection and typically last about a week. PDCoV was first detected in Hong Kong in 2012 (4). Since 2014, it has been widely reported in the United States (5), South Korea (6), Thailand (7), Canada (8), and mainland China (9, 10), causing a substantial financial toll on the pig industry due to its 30%–80% mortality rate and the reduced growth rates in newborn piglets (11). In 2021, PDCoV was even detected in plasma samples from children in Haiti, highlighting its potential for interspecies transmission (12). Therefore, the development of a rapid and sensitive method to monitor PDCoV epidemics in pig herds is vitally important to prevent its spread.

The spike (S) protein of PDCoV is a multifunctional molecule that mediates viral entry into its host cells. It initiates infection by binding to host cell receptors via the S1 subunit, after which the S2 subunit facilitates membrane fusion between the virus and host cell (13). Aminopeptidase N (APN) is recognized as the cellular receptor for PDCoV, and the key amino acid residues Y316, K379, E426, and W429 in the APN are crucial for the cross-species transmission of PDCoV. In addition to mediating viral entry, the S protein is a major inducer of the host immune responses (14). It has been established that the S protein is the most important ingredient in inactivated vaccines and a key target in vaccine design. It is also an important target for virus detection and the early diagnosis of infection.

Multiple methods have been developed to detect PDCoV infection, which can be classified as molecular or immunologic methods. The molecular methods include the TaqMan-based real-time reverse transcription-PCR (RT-PCR) assay (15, 16), SYBR Green I-based duplex quantitative PCR assay (17), duplex nested RT-PCR (18), and a CRISPR/Cas13a-based rapid detection method (19). All of these methods detect viral nucleic acid. However, the tedious operation and heavy workload of RNA extraction make its large-scale detection difficult. Furthermore, the sensitivity of detection largely depends on skilled personnel and sophisticated equipment, the quality of the sample, and the specificity of the primers used, without which false-positive results can occur. These conditions limit the application of these methods in the clinical context. The most commonly used immunoassay for PDCoV detection is the enzyme-linked immunosorbent assay (ELISA) (20–22). ELISA has been widely used in the diagnosis of human and animal diseases because its advantages include its simple operation, strong specificity, and high sensitivity (23, 24). Among various ELISA methods, double-antibody sandwich enzyme-linked immunosorbent assay (DAS-ELISA) can be used to identify early viral infections by detecting viral antigens in samples. However, despite the excellent performance of DAS-ELISA in terms of detection efficiency and accuracy, the complexity of antibody preparation and the potential reduction in sensitivity in cases of multiple infections are undeniable drawbacks to its application (25). These limitations must be overcome by improving experimental methods and optimizing detection procedures to ensure reliable diagnostic outcomes.

Studies have demonstrated the cross-reactivity of the N proteins of PDCoV and PEDV (26), which can reduce the specificity of diagnostic assays that rely on the N protein as the marker for detection. Therefore, in this study, the PDCoV S protein was expressed in the ExpiCHO expression system, and the purified S protein was then used as an immunogen to immunize mice to generate monoclonal antibodies. Compared with prokaryotic expression systems, the CHO expression system allows the correct folding of proteins, provides accurate post-translational modifications, such as glycosylation and acetylation, and expresses proteins with natural activities. Moreover, the purification of proteins is less challenging in the CHO expression system. A novel DAS-ELISA was then established with monoclonal antibody (MAb) 3D7 as the capture antibody and horseradish peroxidase (HRP)-labeled MAb 9G4 as the detection antibody. The DAS-ELISA has high sensitivity, specificity, and reproducibility and is, therefore, a reliable method for the detection of the PDCoV antigen in clinical samples and the evaluation of the S protein content of inactivated or subunit vaccines.

## MATERIALS AND METHODS

### Cells and viruses

ExpiCHO-S cells (Thermo Fisher Scientific catalog number A29133), SP2/0 (Biodragon, catalog number BDXB-0001), and LLC-PK (porcine kidney, American Type Culture Collection no. CL-101) are maintained in our laboratory. The ExpiCHO-S cells were cultured in ExpiCHO stable production medium (Gibco, USA). The LLC-PK cells were cultured in a minimum essential medium (MEM; Gibco, USA) containing 10% fetal bovine serum (FBS; BBI Life Sciences, China). The SP2/0 and hybridoma cell strains were cultured in Dulbecco’s minimum essential medium (Gibco) containing 20% FBS. The PDCoV strain (GenBank accession no. MN064712) and PEDV strain (GenBank accession no. MH816969.1) are maintained in our laboratory. TGEV-positive and PoRV-positive samples were collected from farms and confirmed with RT-PCR (Jonln, catalog number JL-T1880). LLC-PK cells in MEM containing 10 µg/mL trypsin were used to propagate PDCoV.

### Production and purification of MAbs against S protein

The ectodomain of the S gene amplified from PDCoV strain CH/XJYN/2016 was inserted into the pCDNA3.1(+) eukaryotic expression vector. The plasmid was diluted to 1 mg/mL with OptiPRO medium, and then the diluted plasmid was mixed with ExpiFectamine CHO transfection reagent at a ratio of 1:4. The mixture of the plasmid and transfection reagent was incubated at room temperature for 5 min and then added to cultured CHO cells. The S protein expressed was collected after 8 days and purified with Ni-NTA agarose (Qiagen, Germany). To generate MAbs, 6–8-week-old female BALB/c mice were each primed with 20 µg of purified S protein via the subcutaneous route. Complete Freund’s adjuvant was used for the first immunization, and incomplete Freund’s adjuvant was used for booster immunizations at 14 day intervals. The adjuvant and purified S protein were mixed and emulsified in a volume ratio of 1:1. No adverse reactions were observed after immunization of mice. After three rounds of immunization, the spleen cells of the mice were extracted and fused with SP2/0 cells according to the protocol of Köhler and Milstein (27). After 7 days, plates coated with S protein or PDCoV were used to screen for positive samples. Double-positive samples were subcloned twice with limiting dilution and injected into BALB/c mice treated with Freund’s incomplete adjuvant. The ascites fluid of the mice was collected and purified with Protein G Agarose Resin 4FF (Yeasen, China) to obtain the MAbs. The purity of the purified MAbs was confirmed with SDS-PAGE, and the titers were determined with an indirect ELISA. The antibody subtypes were identified according to the manual of a monoclonal antibody subclass identification enzyme kit (Biodragon, China).

### Indirect ELISA

The purified S protein was diluted to 1 µg/mL with carbonated coating buffer (pH 9.6) and used to coat a 96-well microplate (Corning, USA) overnight at 4°C with 100 µL/well. The purified virus was diluted to 5 µg/mL, and all other conditions were the same as for the S protein. The plates were washed three times with phosphate-buffered saline (PBS) containing 0.05% Tween 20 (PBST) and blocked with 5% bovine serum albumin (BSA) for 2 h at 37°C. To screen for positive hybridomas, the supernatants of the hybridoma cells were diluted 1:5 in PBST, and 100 µL was then added to each well. The plates were incubated for 45 min at 37°C. After the samples were washed, HRP-conjugated goat anti-mouse IgG (Abcam, UK), diluted 1:10,000 in PBST, was added (100 µL/well), and the samples were incubated for 40 min at 37°C. After the samples were washed, 100 µL/well of freshly prepared 3,3′,5,5′,-tetramethyl benzidine substrate solution was added to each sample. The reaction was performed for 10 min at 37°C in the dark and then stopped by the addition of 2 M H_2_SO_4_ (100 µL/well). The optical density at a wavelength of 450 nm (OD_450_) was measured with a microplate reader.

The S protein was used for coating to determine the antibody titers, and the coating concentration and the indirect ELISA procedure were as described above. The monoclonal antibodies were serially diluted twofold starting from a 1:100 dilution. Mouse IgG was used as the negative control, and samples with an OD_450_ value greater than 2.1 times that of the negative control were considered positive.

### Immunofluorescence assay

LLC-PK cells were seeded in six-well plates, grown to confluence, and then infected with PDCoV at a multiplicity of infection of 0.001 for 1 h. The inoculum was discarded, and fresh MEM containing 10 µg/mL trypsin was added to the cells. At 36 h post-infection (hpi), the cells were incubated with 4% paraformaldehyde for 1 h and permeabilized with 0.1% Triton X-100 for 10 min at room temperature. The plates were then washed three times with PBS and incubated with the purified MAbs for 12 h at 4°C. After washing, the cells were incubated with 4,6-diamidino-2-phenylindole (Abcam; diluted 1:2,000, 1 mL/well) and then with an Alexa-Fluor-488-conjugated anti-mouse IgG secondary antibody (Abcam; diluted 1:2,000, 1 mL/well) for 1 h at 37°C in the dark. The samples were washed three times with PBS, and then 1 mL of PBS was added to them. Fluorescence was observed with a fluorescence microscope (Olympus, Japan).

### Establishment and optimization of DAS-ELISA

Checkerboard titrations were used to establish the optimal dilution rate for the detection antibody and the ideal concentration of the capture antibody. The capture antibody, MAb 3D7, was diluted in carbonated coating buffer (pH 9.6) at concentrations of 1, 2, 4, 6, 8, 12, 16, and 20 µg/mL. The HRP-labeled MAb 9G4 was diluted in PBST in ratios of 1:200, 1:400, 1:800, 1:1,000, 1:2,000, and 1:4,000. Aliquots (100 µL) were added to each well and incubated for 40 min at 37°C. The optimal concentration of the capture antibody was determined by calculating the highest positive-to-negative (P/N) ratio after MAb 3D7 was coated onto the plates and incubated for 1, 2, 3, or 4 h at 37°C or 12 h at 4°C. The blocking conditions were tested using 1%, 3%, or 5% (wt/vol) BSA, 5% (wt/vol) skimmed milk in PBST, or a liquid plate sealer, and the plates were incubated at 37°C for 1 h. The optimal blocking time was assessed for 1, 2, 3, and 4 h at 37°C or 12 h at 4°C. The incubation time for the detection antigen (in PDCoV-infected or PEDV-infected culture supernatants) was tested for 0.5, 1, 1.5, or 2 h at 37°C or 12 h at 4°C. The incubation time for HRP-labeled MAb 9G4 was tested for 30, 45, 60, 75, 90, and 105 min at 37°C. Finally, development times of 5, 10, 15, and 20 min were tested. All experimental conditions were evaluated based on the P/N ratio.

### Determination of the cut-off value of DAS-ELISA

A total of 56 PDCoV-negative anal swabs were obtained from healthy piglets. The anal swabs were diluted with 1.5 mL of PBS, vortexed for 1 min, and inactivated for 30 min at 56°C. After centrifugation at 12,000 × *g* for 3 min, the supernatants were collected in 1.5 mL microcentrifuge tubes. The samples were analyzed with the established DAS-ELISA under the previously determined optimal conditions. The optical density (OD) was measured at 450 nm. The cut-off value was calculated with the formula *X* + 3SD, where *X* is the mean value for the 56 negative samples, and SD is the standard deviation.

### Specificity, sensitivity, and reproducibility

PEDV, TGEV, and PoRV cause clinical symptoms similar to those of PDCoV infection and were therefore used to verify the specificity of the DAS-ELISA. Anal swabs that tested positive for one or other of the four viruses were processed with an established protocol and confirmed with the DAS-ELISA. The purified S protein and PDCoV-infected culture supernatants were serially diluted and used to detect the sensitivity of the DAS-ELISA. To test the reproducibility of the DAS-ELISA, we performed intra-assay replicate tests and comparisons following the steps of the established method. Eight anal swabs from within the same batch of plates were tested, and the intra-assay coefficient of variation (CV) was calculated. Eight anal swabs from different batches of plates were tested, and the inter-assay CV was calculated.

### Comparison of DAS-ELISA and RT-PCR

RT-PCR is the gold-standard technique for the diagnosis of PDCoV infections and is widely used for the detection of many gastrointestinal pathogens (28). To verify the consistency between the results of the established DAS-ELISA and RT-PCR, 145 anal swabs collected from different farms were analyzed with both the DAS-ELISA and RT-PCR. The anal swabs were treated as described above, and RNA was extracted from 200 µL of supernatant and analyzed with RT-PCR, using the primers (forward) 5′-ACGTCGTAAGACCCAGCATC-3′ and (reverse) 5′-CCCACCTGAAAGTTGCTCTC-3′. The thermal cycling conditions were predenaturation at 95°C for 2 min, followed by 40 cycles of denaturation at 95°C for 15 s, and annealing/extension at 56°C for 1 min. The consistency of the DAS-ELISA and RT-PCR was evaluated from the coincidence rate and the kappa (*κ*) value (29).

### Preparation and detection of inactivated viral antigen

The virus titer was determined with TaqMan fluorescence quantitative PCR before virus inactivation (30, 31). Two methods were used to inactivate the PDCoV virus. In the first method, the virus was treated with 0.1% (vol/vol) formaldehyde and heated in a water bath for 24 h at 37°C. In the second method, the virus was inactivated with 1% (vol/vol) 0.2 M binary ethylene imine (BEI) for 24 h at 30°C. Both live and inactivated viruses were detected with the DAS-ELISA. The preparation method for the 0.2 M BEI solution involved the following steps. Bromoethylamine hydrobromide (BEA, 4.1 g) was aseptically weighed, added to 100 mL of 0.2 M NaOH solution, and its complete dissolution ensured. The solution was placed in a 37°C water bath for 1 h and mixed thoroughly every 15 min. During this time, BEA cyclized to form BEI. When using BEI for virus inactivation, it is crucial to wear personal protective equipment to prevent direct contact with it.

### Statistical analysis

The data were visually displayed with the GraphPad Prism software (version 8.0.2; GraphPad Software, San Diego, CA, USA). Each sample was tested at least three times, and the results are presented as the mean (*X*) ± SD. Reproducibility was evaluated with the CV, and the degree of agreement between different methods was assessed with the kappa value.

## RESULTS

### Preparation and characterization of MAbs

**Fig 1:**
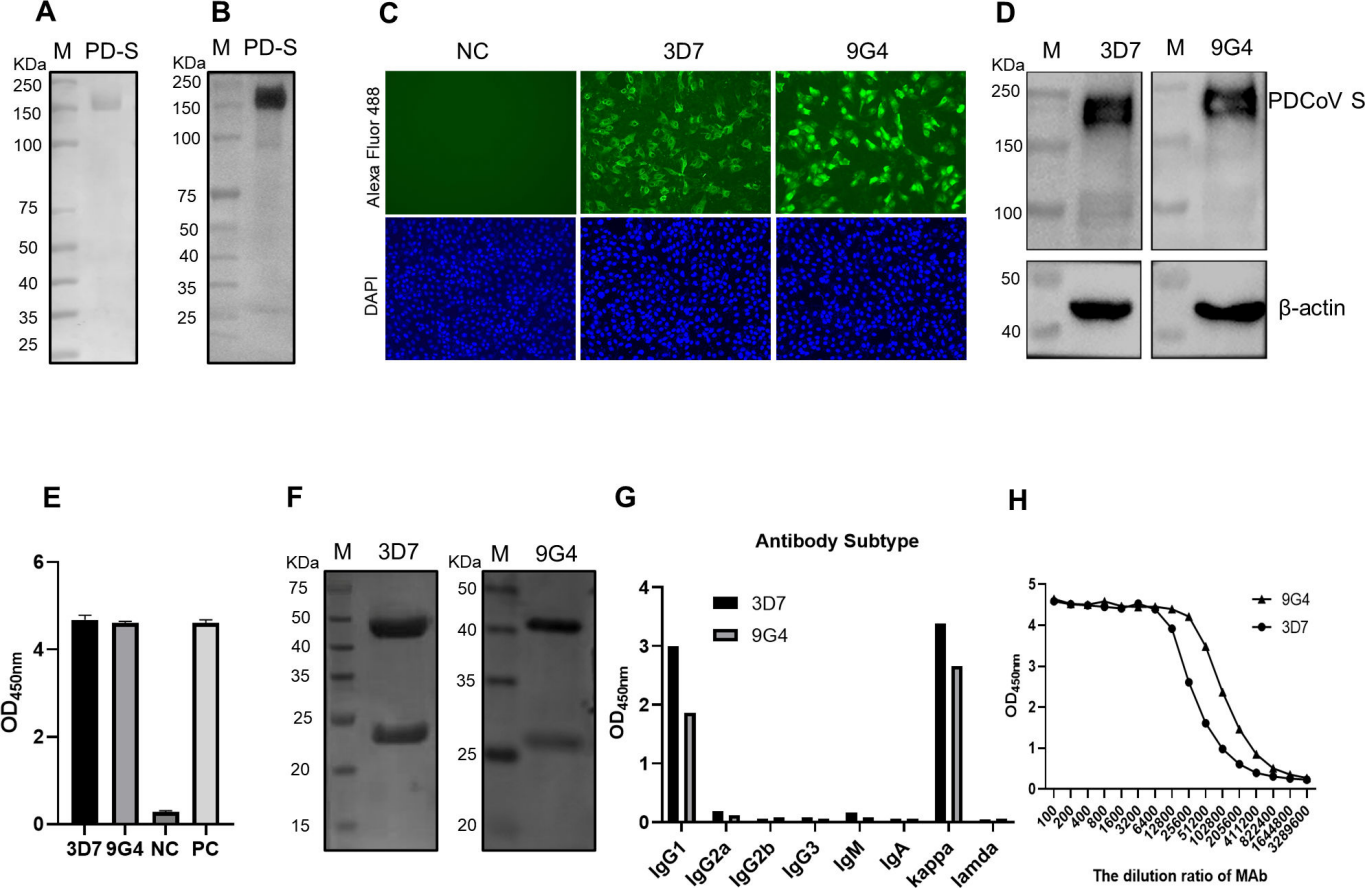
Preparation and characterization of MAbs. Purified S was evaluated with SDS-PAGE (**A**) and western blotting (**B**). The reactivity of the MAbs with PDCoV was confirmed with an immunofluorescence assay (**C**), western blotting (**D**), and indirect ELISA (**E**). (**F**) The purity of the MAbs was determined with SDS-PAGE. (**G**) The antibody subtypes were determined according to the manual for a monoclonal antibody subclass identification enzyme kit. (**H**) The titers of the purified MAbs were determined with an indirect ELISA in 96-well microtiter plates coated with S protein.

ExpiCHO-S cells were transfected with a plasmid expressing the ectodomain of the S protein using a high-titer experimental protocol. The cell culture supernatant was collected, centrifuged, and purified with nickel column affinity chromatography to obtain the recombinant protein, which was confirmed with SDS-PAGE (Fig. 1A) and a western blotting assay using PDCoV-positive porcine serum as the probe (Fig. 1B). To obtain antibodies directed against the S protein, the purified S protein was used to immunize mice. Two specific monoclonal antibodies directed against the S protein of PDCoV (designated 3D7 and 9G4) were obtained with the hybridoma cell fusion technique. The immunoreactivity of the MAbs against the S protein of PDCoV was determined with an immunofluorescence assay (Fig. 1C), western blotting assay (Fig. 1D), and ELISA (Fig. 1E). MAbs 3D7 and 9G4 directed against the PDCoV S protein specifically reacted with PDCoV. The antibodies were purified with Protein G Agarose Resin 4FF, and their purity was determined with SDS-PAGE, which showed clear heavy (~50 kDa) and light (~25 kDa) chains (Fig. 1F). The antibody subtypes of the purified MAbs 3D7 and 9G4 were both IgG1, and the light chains were kappa, according to the OD_450_ values (Fig. 1G). The titers of the purified MAbs were 1:51,200 and 1:205,600, respectively, when determined with an indirect ELISA using 96-well microtiter plates coated with S protein (Fig. 1H).

### Establishment of the DAS-ELISA

**Fig 2:**
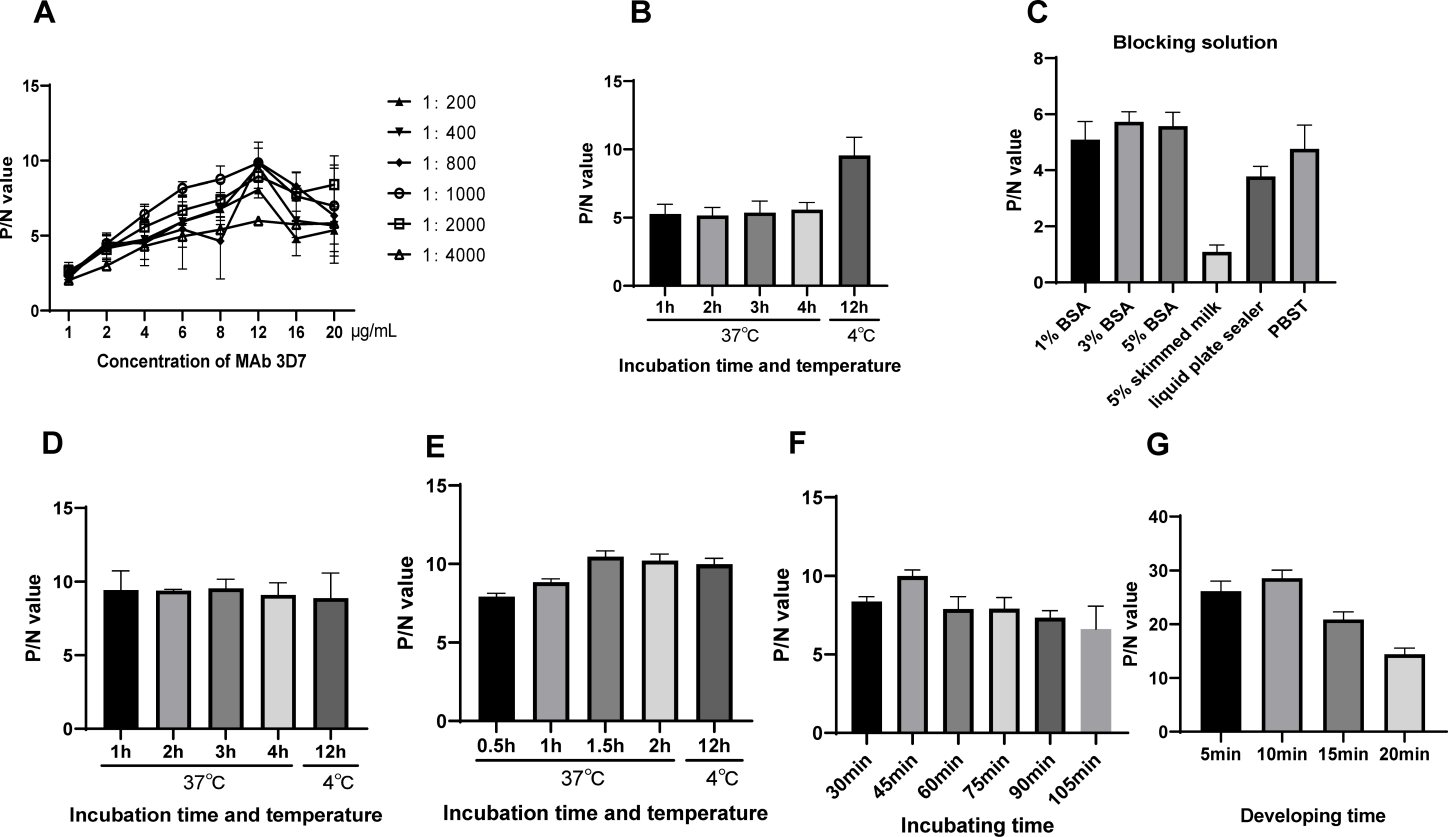
Optimal conditions for the DAS-ELISA. (**A**) The optimal concentration of coating antibody and optimal dilution of the capture antibody were determined with the checkerboard method. (**B**) Optimum coating time and temperature. Optimum blocking buffer (**C**), incubation time, and temperature (**D**). Optimum reaction time of antigen (**E**) and detection antibody (**F**). (**G**) Optimum development time. PDCoV-infected or PEDV-infected culture supernatants were analyzed as the positive and negative controls. All experimental conditions were evaluated based on the P/N ratio.

To establish the DAS-ELISA, the optimal reaction conditions for the capture MAb 3D7 and HRP-labeled MAb 9G4 were determined with checkerboard titrations. The results indicated that a concentration of 12 µg/mL for the capture antibody paired with a 1:1,000 dilution of the detection antibody most effectively detected PDCoV (Fig. 2A). The optimal conditions for coating MAb 3D7 onto ELISA plates were identified as 12 h at 4°C (Fig. 2B). The blocking buffer and blocking time were optimized, and a 3% BSA blocking solution yielded the highest P/N ratio (Fig. 2C). However, no significant differences were observed between different blocking times (Fig. 2D). Therefore, a blocking period of 1 h was used in subsequent experiments. We also determined the optimal binding time between the PDCoV antigen and the detection antibody. The results indicated that a binding time of 1.5 h was optimal for the PDCoV antigen (Fig. 2E), and the ideal reaction time for the detection antibody was 45 min (Fig. 2F). Finally, the development time was assessed, and the highest P/N ratio was achieved after development for 10 min (Fig. 2G).

### Determination of the cut-off value of DAS-ELISA

**Fig 3:**
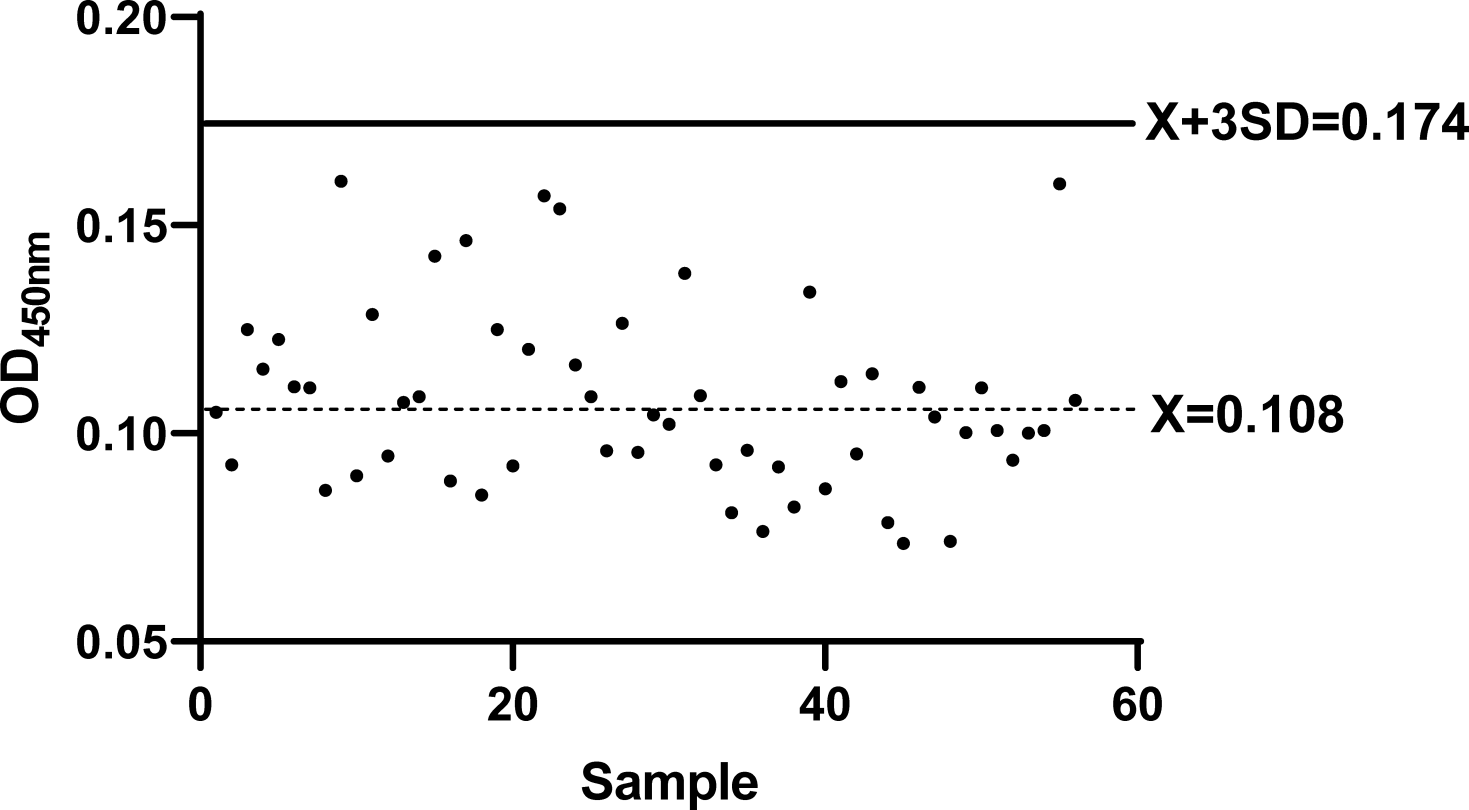
The cut-off value of the DAS-ELISA. Fifty-six PDCoV-negative anal swab samples were used to establish the cut-off value.

A total of 56 PDCoV-negative anal swab samples were used to establish the cut-off value, as shown in Fig. 3. With a mean (*X*) of 0.108 and an SD of 0.022, the determined cut-off was set at 0.174. Therefore, the samples were classified as positive if their OD_450_ value exceeded 0.174 and negative if the value was ≤0.174.

### Specificity, sensitivity, and reproducibility of DAS-ELISA

**Fig 4:**
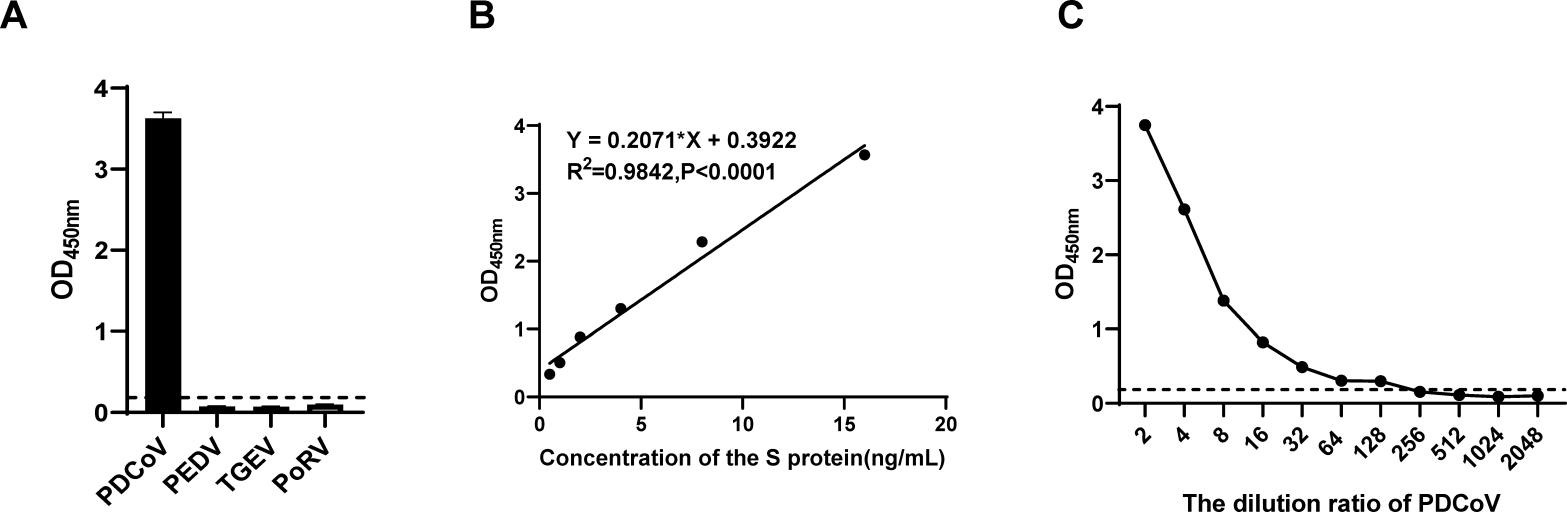
Specificity and sensitivity of the DAS-ELISA. (**A**) Samples positive for PDCoV, PEDV, TGEV, or PoRV were analyzed with the DAS-ELISA to verify its specificity. (**B**) Standard curve of the DAS-ELISA. (**C**) Sensitivity of the DAS-ELISA for PDCoV.

To determine the specificity of the DAS-ELISA, samples positive for PDCoV, PEDV, TGEV, or PoRV were analyzed. The results showed that the OD_450_ value was 3.628 for PDCoV-positive anal swabs, whereas it was lower than the cut-off value (0.174) for the other viruses (Fig. 4A). These results suggested that the DAS-ELISA had good specificity for the detection of PDCoV and did not cross-react with PEDV, TGEV, or PoRV.

To evaluate the sensitivity of the DAS-ELISA, serial twofold dilutions of purified S protein (128 ng/mL) and PDCoV-infected culture supernatants (1 × 10^6.4^ copies/μL) were prepared on an ELISA plate. A standard curve was constructed, correlating the OD_450_ values with the concentration of purified S protein, defined by the linear equation *y* = 0.2071*x* + 0.3922, with an *R*² value of 0.9842 (Fig. 4B). This methodology showed a good linear relationship between the concentration of S protein and OD_450_ value within a range of 0.5–16 ng/mL, achieving a sensitivity threshold of 0.12 ng/mL. For PDCoV, a dilution of 1:128 (1.96 × 10^3^ copies/μL) produced an OD_450_ of 0.29, exceeding the cut-off value, indicating a detection limit of 1.96 × 10^3^ copies/μL (Fig. 4C).

To determine the reproducibility of the DAS-ELISA, intra-assay reproducibility was evaluated by conducting three replicate analyses of eight PDCoV-positive anal swab samples within a single batch. Inter-assay reproducibility was evaluated by performing the DAS-ELISA across three separate batches. As shown in Table 1, the intra-assay CV was <10%, and the inter-assay CV was <10%, indicating the good reproducibility of the DAS-ELISA.

**TABLE 1:** The results of intra-batch and inter-batch duplicability tests

	Sample	Times	Average	SD	CV%
Intra-assay	1	3	1.18	0.03	2.22
	2	3	2.49	0.09	3.48
	3	3	4.01	0.28	6.88
	4	3	3.93	0.15	3.71
	5	3	3.33	0.14	4.14
	6	3	3.76	0.17	4.58
	7	3	1.09	0.05	4.21
	8	3	3.90	0.07	1.69
Inter-assay	1	9	1.21	0.06	4.67
	2	9	2.51	0.06	2.43
	3	9	4.00	0.15	3.83
	4	9	4.04	0.06	1.52
	5	9	3.48	0.22	6.27
	6	9	3.65	0.15	4.07
	7	9	1.05	0.05	5.06
	8	9	3.99	0.06	1.55

### Field sample analysis

A total of 145 anal swab samples were collected from different farms. As shown in Table 2, nine samples tested negative for PDCoV with the DAS-ELISA but were positive on RT-PCR, and four samples tested positive on the DAS-ELISA but were negative on RT-PCR. Among the total 145 anal swab samples, 52 were positive and 80 were negative, as determined using these two methods. The accuracy of these two detection methods was 91.03%, and the *κ* value was 0.814, suggesting high consistency between the DAS-ELISA and RT-PCR methods.

**TABLE 2:** Comparison of DAS-ELISA and RT-PCR for the detection of PDCoV in anal swabs

DAS-ELISA					
	Anal swabs	Positive	Negative	Total	Coincidence rate
RT-PCR	Positive	52	9	61	91.03%
	Negative	4	80	84	
	Total	56	89	145	
	Kappa	0.814			

### Detection of inactivated viral antigen

**Fig 5:**
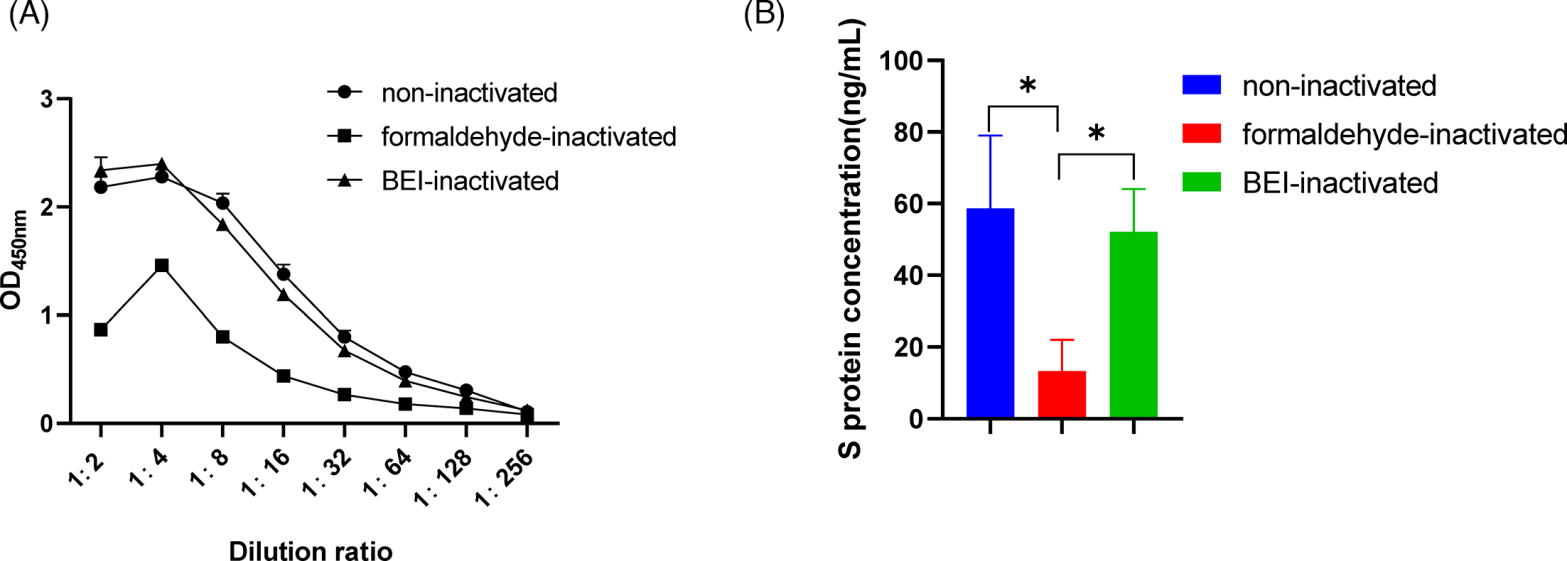
Detection of inactivated viral antigens. (**A**) Live and inactivated viruses were analyzed with the DAS-ELISA by measuring OD_450_ value. (**B**) The concentration of S protein differed with different inactivation methods. The concentration of S protein was calculated from the OD_450_ and the previously established standard curve.

The content and structural integrity of the S protein are closely related to the immune efficacy of inactivated vaccines and are important data when evaluating vaccine quality. To determine whether the method we established can detect the S protein after viral inactivation, we inactivated PDCoV with formaldehyde or BEI. The results are shown in Fig. 5. The DAS-ELISA still detected the S protein after PDCoV inactivation with formaldehyde or BEI. When we compared the results for the inactivated virus with those for the non-inactivated virus, the formaldehyde inactivation method seemed to cause greater damage to the integrity of the S protein than BEI (*P* < 0.05). The S protein content in the BEI inactivation group was essentially the same as that in the non-inactivated group (*P* > 0.05).

## DISCUSSION

PDCoV is an emerging coronavirus with a global distribution, primarily transmitted via the fecal-oral route, causing diarrhea and dehydration in sows and the acute death of newborn piglets. More importantly, it has the potential for cross-species transmission, infecting various animals, including chicks and calves (32, 33), and even posing a serious threat to human health (12). Since the outbreak in 2014, PDCoV has caused significant losses to the global swine industry. However, no effective vaccine has yet been developed, although accurate and clinically practical diagnostic methods are extremely important for the early detection and control of PDCoV.

As a commonly used detection method, ELISA has the advantages of being fast, accurate, easy to operate, and less restricted by the need for clinical equipment, and it is widely used for the diagnosis of clinical diseases. Several indirect ELISAs based on the PDCoV S1 or N protein have been developed to detect PDCoV infection (20). Compared with indirect ELISA, DAS-ELISA offers greater sensitivity and specificity, particularly in detecting antigens in blood and oral swabs, whereas indirect ELISA tends to be less sensitive. The DAS-ELISA uses an antibody directed against a pathogen antigen to detect infection in animals and has been used to detect PEDV, swine acute diarrhea syndrome coronavirus, and avian influenza virus (34–36). The results of this method are highly consistent with those of RT-PCR. Several studies have established double-antibody sandwich ELISAs for detecting PDCoV infections based on a rabbit polyclonal antibody or a mouse monoclonal antibody directed against the PDCoV N protein (21, 37). However, cross-reactivity between the N proteins of PDCoV and PEDV, together with the high coinfection rate of PDCoV and PEDV in clinical samples, may increase the difficulty of the differential diagnosis of PDCoV and PEDV, leading to false-positive results (26). Furthermore, because the N protein is located inside the viral particle envelope, antibodies directed against N can only detect incomplete viral particles, which may reduce the sensitivity of detection.

The S protein of PDCoV is located on the viral envelope and plays a crucial role in the invasion of host cells by binding to receptor molecules on them (13). It contains multiple neutralizing epitopes, making it a key target for vaccine design. In inactivated vaccines, the content of S protein is an important indicator of the vaccine’s immunogenicity. The S protein is also often used as a detection target to monitor the infection status of the coronavirus. For example, studies have used two different monoclonal antibodies targeting the S protein to develop a colloidal gold-based detection technology for Middle Eastern respiratory syndrome coronavirus infections (38). Other research has used an anti-PEDV S monoclonal antibody as the capture antibody to immobilize PEDV to develop an ELISA for detecting PEDV IgA antibodies in milk (39). Nguyen and colleagues developed an antibody-coupled graphene for the detection of viruses, which detected the severe acute respiratory syndrome coronavirus 2 spike protein with detection limits of 1 fg/mL in PBS and 3.75 fg/mL in saliva (40). There have been numerous reports of the use of antibodies targeting the S protein to determine the content of S protein for the diagnosis of early-stage coronavirus disease 2019 infections (41–45).

The DAS-ELISA method we established utilizes two distinct monoclonal antibodies directed against the PDCoV S protein as paired antibodies, thus avoiding the inter-batch variability associated with the use of polyclonal antibodies and lengthy production cycles. The fusion and screening of the monoclonal antibodies were performed according to the preparation processes used for related antibodies, including those directed against PEDV and ASFV (46, 47). Seven days after fusion, the wells were screened for PDCoV S protein and the whole PDCoV virus coating the wells. Doubly positive samples in the wells were subcloned to obtain the monoclonal antibodies. By dual screening for the whole virus and S protein, the specificity of the assay was ensured. Throughout the entire production process, no issues affecting the stability or preparation efficiency of the antibodies were encountered. The expression efficiency and activity of the antibodies were also very high compared with those reported in previous studies (47). Monoclonal antibodies can be produced in large amounts in eukaryotic cells, which allows their efficient mass production, and the process of purifying them is relatively simple.

The method developed here displayed good sensitivity and specificity. The detection limits of the developed DAS-ELISA were 0.12 ng/mL of purified S protein and 1.96 × 10^3^ copies/μL PDCoV. Specificity assays showed that the developed DAS-ELISA does not cross-react with other swine enteric coronaviruses, such as PEDV, TGEV, and PoRV, which are similar to PDCoV in their clinical symptoms. To verify the consistency between the results of this DAS-ELISA and RT-PCR, 145 anal swabs collected from different farms were analyzed with both methodologies. The concordance rate was 91.03%, with a *κ* value of 0.814, indicating that this DAS-ELISA is a reliable method for the detection of PDCoV in clinical samples. This method can also be used to quantitatively assess the S protein content in inactivated and subunit vaccines, providing a powerful tool for the evaluation of these vaccines. Therefore, the DAS-ELISA established in this study should be a reliable and effective tool for detecting PDCoV infection and the efficacy of PDCoV vaccines.

## Data Availability

The data supporting the results of this study are available from the corresponding author upon reasonable request.
